# Orange Peel *Lactiplantibacillus plantarum*: Development of A Mucoadhesive Nasal Spray with Antimicrobial and Anti-inflammatory Activity

**DOI:** 10.3390/pharmaceutics16111470

**Published:** 2024-11-18

**Authors:** Elisa Corazza, Asia Pizzi, Carola Parolin, Barbara Giordani, Angela Abruzzo, Federica Bigucci, Teresa Cerchiara, Barbara Luppi, Beatrice Vitali

**Affiliations:** 1 Drug Delivery Research Laboratory, Department of Pharmacy and Biotechnology, Alma Mater Studiorum, University of Bologna, Via San Donato 19/2, 40127 Bologna, Italy; elisa.corazza7@unibo.it (E.C.); angela.abruzzo2@unibo.it (A.A.); federica.bigucci@unibo.it (F.B.); teresa.cerchiara2@unibo.it (T.C.); 2 Beneficial Microbes Laboratory, Department of Pharmacy and Biotechnology, Alma Mater Studiorum, University of Bologna, Via San Donato 19/2, 40127 Bologna, Italy; asia.pizzi2@unibo.it (A.P.); carola.parolin@unibo.it (C.P.); barbara.giordani4@unibo.it (B.G.); b.vitali@unibo.it (B.V.)

**Keywords:** *Lactiplantibacillus plantarum*, antimicrobial activity, anti-inflammatory activity, reconstituted spray, nasal delivery

## Abstract

**Background/Objectives:** Due to the high frequency and severity of upper respiratory bacterial infections, probiotics could offer a new medical approach. We explored the antibacterial and anti-inflammatory properties of the new strain *Lactiplantibacillus plantarum* BIA and formulated a nasal spray. **Methods:** *L. plantarum* BIA was isolated from orange peel and taxonomically identified through 16S rRNA gene sequencing. Its antibacterial activity was tested against *Pseudomonas aeruginosa*, *Streptococcus pyogenes*, *Bacillus subtilis*, *Escherichia coli*, and *Staphylococcus aureus*, while anti-inflammatory potential was evaluated by Griess assay. BIA genome was fully sequenced and analyzed to assess its safety. BIA was formulated in a freeze-dried matrix, containing prebiotics and cryoprotectants, to be reconstituted with a polymer solution. Solutions containing two types of hydroxypropyl methylcellulose (HPMC) and hyaluronic acid were evaluated as resuspending media and compared in terms of pH, viscosity, and mucoadhesion ability. The biological activity of BIA formulated as nasal spray was verified together with the stability of the selected formulations. **Results:** *L. plantarum* BIA inhibited human pathogens’ growth and showed anti-inflammatory activity and a safe profile. In the best-performing formulation, the probiotic is lyophilized in 10% fructooligosaccharides, 0.1% ascorbic acid, and 0.5% lactose and reconstituted with HPMC high viscosity 1% *w*/*v*. This composition ensured the probiotic’s viability for up to six months in its dried form and one week after reconstitution. It also allowed interaction with the nasal mucosa, preserving its antimicrobial and anti-inflammatory activities. **Conclusion:** The developed nasal spray could become a promising formulation in the field of nasal infectious and inflammatory diseases.

## 1. Introduction

The occurrence of bacterial infections in the nasal cavity and nasopharynx is quite common and should not be neglected, as pathogens can further colonize the lower respiratory tract, namely the trachea, the smaller airways (bronchi and bronchioli), and the alveoli [1], possibly leading to severe respiratory infections. However, the resident microbiota of the upper respiratory tract can prevent pathogens from establishing an infection on the mucosal surface, thus assuring protection against colonization. In particular, it has been reported that lactobacilli present in the nasal cavity can protect the epithelial layer and maintain immune homeostasis [2], representing an important shield against microbial infections. In a complementary way, the nasal delivery of probiotics can strengthen the protective activity offered by the nasal microbiota, serving as both a preventive strategy to inhibit the first step of the pathogenesis of respiratory infections and a treatment approach to reduce pathogens’ density at the mucosal site [3]. This evidence, along with the awareness that traditional treatments can be complicated by widespread antibiotic resistance [4], opens up the exploration of new medical approaches.

Lactic acid bacteria (LAB) and their metabolites are extensively used as probiotics and natural preservatives in both food and medicine industries [5]. Many probiotics have demonstrated a range of beneficial properties, including antimicrobial activity, which is exerted through the inhibition of the growth, colonization, and development of pathogenic microorganisms. This antagonism towards pathogens is associated with different mechanisms, i.e., competition for nutrients and adhesion sites, induction of environmental changes that can be harmful to pathogens, and production of antimicrobial substances [6]. This project aims to formulate a nasal spray that can deliver probiotics to the nasal cavity to maintain respiratory health, as respiratory tract infections are one of the sources of incidence and mortality in the world [7]. The increase in drug-resistant microbes, combined with the lack of available vaccines against many respiratory pathogens, is making the effective treatment of respiratory infections highly challenging. In light of this, lattobacilli probiotics have recently been explored as a therapeutic option for various bacterial and viral infections of the respiratory tract [8,9,10,11].

Recently, LAB have also gained wide recognition for their critical role in modulating inflammatory responses, including the regulation of cytokine production, enhancement of intestinal barrier function, and activation of immune cells [12]. They also produce metabolites such as short chain fatty acids (SCFAs) that can modulate host respiratory immunity [13]. SCFAs exhibit numerous immunomodulatory properties, including the differentiation and promotion of T-regulatory cells, which subsequently reduce inflammation [14]. The mechanisms underlying the health-promoting effects of *Lactobacillus* probiotics on the respiratory tract are complex, but numerous studies have been conducted to identify probiotics capable of treating respiratory diseases. For example, the administration of certain *Lactobacillus* species has been shown to alleviate symptoms of respiratory conditions, such as airway infections, asthma, lung cancer, and cystic fibrosis, in both animal models and clinical trials [15].

In the present study, we isolated for the first time a new strain of *Lactiplantibacillus plantarum,* i.e., *L. plantarum* BIA, from orange peel with noteworthy antibacterial and anti-inflammatory properties. *L. plantarum* is one of the most extensively studied LAB species due to its potent probiotic properties [16]; furthermore, owing to its high acid tolerance and antagonistic activity against pathogens, it is also able to survive in harsh environments [17]. This species is classified as Generally Recognized as Safe (GRAS) according to the U.S. Food and Drug Administration due to its long history of safe use in fermented foods and its presence in the normal intestinal and urogenital microbiota of humans [18]. It also possesses the qualified presumption of safety (QPS) status from the European Food Safety Authorities (EFSA), and thanks to these fulfilments, several studies explore the beneficial potential of probiotic strains belonging to this species [19,20,21,22]. It is known that this species colonizes the healthy human intestinal mucosa, and it is also commonly found in fermented food and vegetables [23,24]. Recently, in-depth research has been dedicated to investigating the probiotic potential of different strains of *L. plantarum* isolated from vegetables and fermented food, as they have demonstrated inhibitory effects on pathogens’ growth, as well as suppression of proinflammatory cytokines [24,25]. Due to their non-invasiveness and ease of self-administration, sprays are widely used for local delivery of active ingredients at the nasal site [26]. However, a careful development of these products is mandatory to ensure their stability over the shelf life and effectiveness following administration. The nature of the formulation, the features of the active ingredient, and the concentration required to deliver the correct dose [27] drive the selection of the appropriate excipients. In the specific case of nasal sprays intended for probiotic delivery, the use of some key functional excipients is a prerequisite [28]. Buffers help maintain an environment suitable for probiotic survival and activity, minimize irritation, and ensure comfort upon administration. Mucoadhesive agents are essential to prolonging the contact time with the nasal mucosa, thus enhancing the potential for probiotic colonization. Dispersing agents are necessary to obtain a homogeneous distribution of the probiotic cells in the spray formulation, ensuring a consistent dose for each administration pump. On the other hand, preservatives should be avoided as they can compromise probiotic survival.

In the present study, a reconstituted mucoadhesive suspension has been chosen as a valuable and advantageous solution to deliver *L. plantarum* BIA cells to the upper respiratory tract. The suspension can be easily prepared before administration by mixing lyophilized probiotic cells with a sterile liquid solution containing a mucoadhesive polymer. This technological approach offers various advantages over both aqueous-based and powder-based traditional nasal formulations. Compared to liquid formulations, reconstituted nasal suspensions maintain probiotic cells in a dry state until use, thus significantly enhancing their viability and bypassing the use of preservatives in the aqueous medium. Moreover, as opposed to powder formulations, reconstituted suspensions are generally more comfortable for nasal administration and provide an optimal moisture environment that does not irritate the mucosa and supports both the colonization and the therapeutic effect of probiotics once administered. They can also contribute to more uniform and reliable dosing, ensuring that the correct dose of probiotics is delivered with each administration using standard user-friendly and convenient nasal spray devices [29]. Overall, the process of reconstitution before use combines the stability benefits of dry storage with the comfort and ease of liquid administration.

## 2. Materials and Methods

### 2.1. Materials

Skim milk (SK), fructooligosaccharides (FOS), spray-dried lactose (LCT), and inulin 90% (IN) were purchased from Difco (Detroit, MI, USA), Farmalabor s.r.l (Canosa di Puglia, BT, Italy), Fagron (Quarto Inferiore, BO, Italy), and ACEF s.p.a. (Fiorenzuola d’Arda, PC, Italy), respectively. Ascorbic acid (AA) and yeast extract (YE) were Fluka products obtained from Sigma-Aldrich (Milano, MI, Italy). Hydroxypropyl methylcellulose low-viscosity Methocel™ E50LV Premium 5P (HPMC-LV) was sourced by Sigma-Aldrich (Milano, MI, Italy), hydroxypropyl methylcellulose high-viscosity Benecel™ K100M Pharm (HPMC-HV) was bought from Ashland Industries Europe GmbH (Schaffhausen, Switzerland), and hyaluronic acid (HA) (0.8–1.5 × 10^6^ Da) was supplied by Farmalabor s.r.l. (Canosa di Puglia, BT, Italy). The mucin type-II from porcine stomach and the E-131 blue food coloring were purchased from Sigma-Aldrich (Milano, MI, Italy) and Candi Gestro s.r.l. (Siderno, RC, Italy), respectively. All the salts employed to prepare buffer solutions were ACS Reagents ≥ 99.0% supplied by Sigma-Aldrich (Milano, MI, Italy). The composition of the phosphate-buffered solution (PBS) pH 6.8 was the following: 21.13 mM Na_2_HPO_4_·12H_2_O; 7.35 mM KH_2_PO_4_; 93.94 mM NaCl. The simulated nasal fluid comprised 128.34 mM NaCl, 17.44 mM KCl, and 2.04 mM CaCl_2_ · 2H_2_O. De Man, Rogosa, and Sharpe (MRS) broth, Nutrient Broth (NB), and Trypticase Soy Broth (TSB) were purchased from Difco (Detroit, MI, USA). The L-cysteine was purchased from Merck (Milan, MI, Italy). The GasPak™ was purchased from EZ Anaerobe Container System (Sparks, MD, USA). The DNeasy Blood & Tissue Kit was purchased from Qiagen (Tegelen, Hulsterweg, Netherlands). The Agar was purchased from VWR Chemicals (Radnor, Pennsylvania, USA). The Qubit dsDNA BR assay kit in conjunction with the Qubit 2.0 fluorometer were obtained from Life Technologies (Monza, MI, Italy). The SQK-LSK109 and EXP-NBD196 kits were purchased from Oxford Nanopore Technologies (Oxford, UK). The Dulbecco’s Minimal Essential Medium (DMEM) was purchased from Sigma-Aldrich (St. Louis, MO, USA). The fetal bovine serum (FBS) was purchased from Gibco Life Technologies Corporation (Segrate, MI, Italy). The L-glutamine was purchased from Merck (Saint Louis, MO, USA). The Griess Reagent Nitrite Measurement Kit was purchased from CellSignaling Technology (Danvers, MA, USA). The lipopolysaccharide (LPS) was purchased from Enzo Biochem (Pero, MI, Italy).

### 2.2. Isolation of L. plantarum Strains from Oranges

LAB were isolated from various organic oranges, specifically Washington Navel variety, collected in February 2024 from Trapani in Sicily (Italy). The orange peel was mixed in De Man, Rogosa, and Sharpe (MRS) broth, which was supplemented with 0.05% L-cysteine. Different dilutions of the resulting mixture were plated on MRS agar plates and incubated at 37 °C for 72 h in anaerobic jars containing GasPak™ (GasPak™ EZ Anaerobe Container System, Sparks, MD, USA). Colonies exhibiting diverse morphologies, with varying rod-shaped forms, as observed under the optical microscope (Nikon ECLIPSE E200), were selected and stored as glycerol stocks. The isolates were routinely cultured in MRS broth supplemented with 0.05% L-cysteine under anaerobic conditions at 37 °C.

### 2.3. Taxonomic Identification of the Isolates by 16S rRNA Gene Sequence

Overnight cultures of the LAB isolates (2 mL) were centrifuged (Beckman Coulter, Brea, California, USA) (10 min at 10,000× *g*) to pellet the cells. Genomic DNA was extracted using the DNeasy Blood & Tissue Kit following the “Pretreatment for Gram-positive bacteria” protocol [30]. The complete 16S ribosomal RNA (rRNA) gene, approximately 1.5 kb in length, was amplified using the universal primers 27F and 1492R [31] and then sequenced. The obtained sequences were compared with those available in BLAST (https://blast.ncbi.nlm.nih.gov, accessed on 4 September 2023) to achieve the taxonomical identification of the isolates.

### 2.4. Assessment of In Vitro Antibacterial Activity of Cell-Free Supernatants from L. plantarum Strains

The cell-free supernatants (CFS) of *L. plantarum* strains were obtained by centrifuging the overnight cultures at 5000× *g* for 10 min at 4 °C, followed by filtration through a 0.22 µm membrane (GVS filter technology, Zola Predosa, BO, Italy). The pH of the CFS was measured using a pH meter (pH-metro BASIC 20 CRISON). The antimicrobial activity of the CFS was tested against the following pathogens: *Escherichia coli* ATCC 11105, *Staphylococcus aureus* ATCC 29213 *Pseudomonas aeruginosa* ATCC 10145, *Bacillus subtilis* ATCC 31324, and *Streptococcus pyogenes* DSMZ 0565. The pathogens were cultured in Nutrient Broth (NB), except for *S. pyogenes,* which was cultured in Trypticase Soy Broth (TSB). The assay was conducted in flat-bottom 96-well plates (Corning Incorporated, Kennebunk, ME, USA), with each well containing 100 µL of *L. plantarum* strains CFS, followed by the addition of 100 µL of pathogen suspension (1 × 10^6^ CFU/mL). Control wells contained 100 µL of sterile MRS medium and 100 µL of the same pathogen suspensions. The plates were incubated at 37 °C for 24 h, and pathogen growth was assessed by measuring the absorbance at 600 nm using an EnSpire Multimode Plate Reader (PerkinElmer Inc., Waltham, MA, USA). The growth of the pathogens in the presence of *L. plantarum* strains CFS was expressed as a percentage relative to the absorbance of the corresponding control.

### 2.5. Assessment of In Vitro Antibacterial Activity of Viable Cells of L. plantarum Strains

The antagonistic activity of *L. plantarum* viable cells was assessed following the protocol described by Siroli et al. (2017) [32] against the pathogens listed in Section 2.4. All the pathogens were cultured twice in Nutrient Broth prior to experimentation. In brief, 5 µL of an overnight culture of each *L. plantarum* strain (10^9^ CFU/mL) was spotted on the surface of MRS agar plates and incubated anaerobically at 37 °C for 24 h. Subsequently, 0.1 mL (approximately 10^8^ CFU/mL) of an overnight culture of each pathogen was mixed with 10 mL of NB or TSB soft agar (0.7% agar) and poured over the MRS plates containing *L. plantarum* spots. After a further 24 h incubation at 37 °C, the plates were inspected for pathogen growth and inhibition zones. The inhibition halos were measured from the outer edge of the spots in four directions, and the average radius was calculated. According to the radius of inhibition, the antagonistic activity shown by *L. plantarum* strains was expressed as follows: -, radius 0–5mm; +, radius between 6–14; ++ radius between 14–22 mm; +++, radius >22 mm. 

### 2.6. Genome Sequencing of L. plantarum BIA

The genomic DNA of *L. plantarum* BIA strain was extracted as previously described in Section 2.3, quantified using the Qubit dsDNA BR assay kit in conjunction with the Qubit 2.0 fluorometer (Life Technologies, Monza, MI, Italy) and subsequently sequenced using Oxford Nanopore Whole Genome Sequencing technology. In brief, the genomic library was prepared utilizing the SQK-LSK109 and EXP-NBD196 kits and sequenced on an R9.4.1 flow cell (MIN106D, Oxford Nanopore Technologies, Oxford, UK) with the GridION X5 Nanopore device (Oxford Nanopore Technologies, Oxford, UK). The genome was assembled de novo using the Canu assembler v2.2 (Koren S, Walenz BP, Berlin K, Miller JR, Bergman NH, Phillippy AM. Canu: Scalable and accurate long-read assembly via adaptive κ-mer weighting and repeat separation. Genome Res. 2017;27) and annotated with Bakta v1.4.1 (Schwengers O, Jelonek L, Dieckmann MA, Beyvers S, Blom J, Goesmann A. Bakta: Rapid and standardized annotation of bacterial genomes via alignment-free sequence identification. Microb Genom. 2021;7), achieving a genome coverage of 39.47X. The *L. plantarum* BIA genome was then screened for antibiotic resistance and virulence-associated genes using three different tools, i.e., AMR Finder Plus software (https://www.ncbi.nlm.nih.gov/pathogens/antimicrobial-resistance/AMRFinder/, updated on 15 September 2024), ResFinder 4.6.0 database (https://cge.food.dtu.dk/services/ResFinder/, accessed on 11 November 2024, threshold for identity: 90.0%, minimum length: 60.0%), and Resistance Gene Identifier (RGI 6.0.3) using the Comprehensive Antibiotic Resistance Database (CARD, version 3.3.0; https://card.mcmaster.ca/, accessed on 11 November 2024).

### 2.7. Evaluation of Anti-Inflammatory Activity

A murine macrophage cell line (RAW264.7) was cultured in DMEM supplemented with 10% FBS and 1% L-glutamine. Cells were incubated at 37 °C in a 5% CO_2_ atmosphere. Anti-inflammatory activity was assessed by measuring nitrite levels in the culture medium using the Griess Reagent Nitrite Measurement Kit, which provides an indirect quantification of nitric oxide (NO) by detecting nitrite, nitrate, and nitrosating agents. RAW264.7 cells were seeded into 24-well plates at a density of 1 × 10^5^ cells per well and incubated for 48 h in complete medium. Inflammation was induced by stimulating the cells with 1 µg/mL of LPS from *E. coli*. The cells were concurrently treated with either *L. plantarum* BIA cells, CFS, or freeze-dried and reconstituted cells for 24 h. Following the treatment, 100 µL of the culture medium was mixed with 100 µL of Griess reagent in a 96-well plate, and nitrite concentration was measured spectrophotometrically at 550 nm using an EnSpire Multimode Plate Reader (PerkinElmer Inc., Waltham, MA, USA). Inflammation levels were expressed as a percentage relative to the absorbance of the control group, in which RAW264.7 cells were stimulated with LPS alone (considered 100%).

### 2.8. Freeze-Drying of L. plantarum BIA

Freeze-drying of *L. plantarum* BIA was achieved by exploiting different lyophilization media (LM), whose composition is reported in Table 1**.** LM were obtained by solubilizing all the components in NaCl 0.9% (*w*/*v*), and sterility was ensured by either autoclave or filtration depending on the excipients’ properties. Precisely, SK, LCT, and YE were solubilized in NaCl 0.9% (*w*/*v*), and then the pH of the resulting solution was adjusted to a value of 7 employing NaOH 5 N and finally autoclaved for 30 min at 110 °C. The LM containing the autoclaved components was completed by adding proper volumes of FOS, AA, and IN, which had been previously dissolved in NaCl 0.9% (*w*/*v*) at higher concentrations compared to those used in the final LM and filtered through a syringe filter with a cut-off of 0.22 µm (Cellulose acetate syringe filter, Sanford, FL, USA). Subsequently, 10 mL of each LM was used to resuspend 10^11^ CFU of *L. plantarum* BIA. For freeze-dried samples, the cell suspensions were put at –20 °C overnight and then freeze-dried (Alpha 1-2 LSCbasic, Martin Christ Gefriertrocknungsanlagen GmbH, Osterode am Harz, Germany) at 0.4 mbar and −60 °C for 24 h; the LM containing BIA were stored at +4–8 °C until use.

### 2.9. Cell Viability upon Freeze-Drying

Viable cell counts were determined before and after the freeze-drying process by plating on MRS agar plates and incubating at 37 °C, in anaerobiosis for 24 h to 48 h. The lyophilized cells were resuspended in 5 mL of NaCl 0.9% (*w*/*v*) containing 0.05% L-cysteine and incubated at 37 °C for 1 h before plating.

### 2.10. Polymer Solution Development and Reconstitution of the Freeze-Dried L. plantarum BIA

Different solutions were obtained by dissolving three mucoadhesive agents in sterile PBS pH 6.8. Specifically, HPMC-LV, HA, and HPMC-HV were investigated at different concentrations depending on the macromolecule: 5% for HPMC-LV, 1.5 and 1.75% for HA, and 0.75% and 1% for HPMC-HV. When HPMC-LV and HA were considered, the polymer was added in small fractions to the buffer under stirring to avoid the formation of aggregates of powder and then kept under gentle stirring at room temperature overnight. In the case of HPMC-HV, the buffer was pre-heated at 80 °C before adding the polymer under stirring, then the sample was kept at 80 °C for 10 min. Like HPMC-LV and HA, HPMC-HV solutions were also maintained at room temperature under gentle stirring overnight before use.

Polymer solutions were first screened in terms of pH, viscosity, and mucoadhesion ability; subsequently, the same features were evaluated once they were used to reconstitute the freeze-dried LM in the absence and presence of the probiotic. For reconstitution, 10 mL of polymer solution was simply poured in the vial containing the lyophilized product and manually shaken to ensure that the content was properly mixed.

#### 2.10.1. pH and Viscosity Determination

The pH of the samples was measured using pH indicator strips with a range of 0–14 (Whatman^®^ Panpeha™, Sigma-Aldrich, Milan, MI, Italy). The strip was fully submerged in the liquid sample, and the resulting color was then matched against the color chart provided on the packaging. The viscosity was determined through a rotational viscometer (Visco Star-R, Fungilab S.A., Barcelona, Spain). Approximately 10 mL of the specimen was poured into the testing tube thermostated by a water jacket at 25 °C. The measurement was conducted using the spindle TR 9 at a speed of 200 rpm.

#### 2.10.2. Mucoadhesive Properties

The influence of HPMC-LV, HA, and HPMC-HV used at certain concentrations on the mucoadhesive character of the nasal formulation was evaluated according to two different methods: dripping test and force of mucoadhesion.

##### 2.10.2.1. Dripping Test

The paper-based dripping test resembles that described by Masiuk and collaborators [33]. Specifically, a squared (10 × 10 cm) paper filter was hydrated with 10 mL of an 8% (*w*/*w*) mucin dispersion in a simulated nasal buffer and dried in the open air for 10 min. The test solution was stained with 0.25% (*w*/*w*) of blue food color and placed inside a nasal spray pump. The coated paper filter was positioned perpendicularly to the nasal spray direction and the formulation was sprayed on it. The displacement distance was measured after 30 s upon contact, and the registered value reflects the product’s tendency to drip off after administration and its adhesive behavior.

##### 2.10.2.2. Force of Mucoadhesion

The force of mucoadhesion was measured by exploiting an adapted tensiometer (Krüss 132869; Hamburg, Germany), according to Corazza et al. 2022 [34]. In this case, the measured adhesive forces were those established between the liquid formulation (either the sole polymer solutions or the reconstituted freeze-dried matrices in the absence and presence of the probiotic) and a specimen of porcine respiratory mucosa. The latter was isolated from porcine tissue obtained from a local slaughterhouse (Salumificio Capelli, Bologna, Italy). The nasal septum was carefully removed, and the turbinates were extracted from the nasal cavity using forceps and a scalpel. The mucosal layer was then detached from the underlying cartilage and rinsed with NaCl 0.9% (*w*/*v*). The thus-obtained nasal mucosa was attached to a circular support (0.90 cm in diameter, 0.35 cm thick) using cyanoacrylate adhesive. It was then hydrated for 2 min with the same mucin dispersion described in Section 2.10.2.1 and suspended from the tensiometer spring. Next, 3 mL of each sample was placed into a small beaker (3 cm in diameter). The mucosa was then lowered towards the sample, and upon contact with the formulation’s surface, it was immediately raised. The force required to take apart the tissue from the liquid formulation was measured, and the higher the force, the stronger the mucoadhesiveness of the product.

### 2.11. Stability Study

Considering the impact of LM composition on bacterial cell viability, the intention to keep the composition of the final product as simple as possible, and the mucoadhesive properties of the polymer solution, the stability study was exclusively performed on the formulation obtained by lyophilizing *L. plantarum* BIA with LM1 and LM4 reconstituted with a 1% HPMC-HV solution. The probiotic in its lyophilized form and the sole polymer solution were stored at +4–8 °C for 6 months, during which their features were evaluated at predetermined time points (7, 30, 60, 90, 150, and 180 days). For the freeze-dried probiotic, cell viability and antimicrobial and anti-inflammatory activities were tested; in the case of the polymer solution, pH and viscosity were determined instead. Conversely, all the aforementioned investigations were necessary to prove the stability of the reconstituted nasal spray when stored at +4–8 °C. Upon reconstitution, cell viability maintenance was ascertained through cell counting at different times over 25 days. In this span, antimicrobial and anti-inflammatory properties were also checked along with pH and viscosity, which were verified at the beginning and the end of the storage period.

### 2.12. Statistical Analysis

The results are reported as mean values accompanied by their respective standard deviations (SD), calculated using data obtained from two or three independent experiments. Statistical analysis was performed on all experimental data using a *t*-test and 1-way Anova, with significance established for *p*-values below 0.05.

## 3. Results and Discussion

### 3.1. Taxonomical Identification of the Strains Isolated from Organic Oranges

Sicilian organic oranges of the Navel variety were employed as a source for the isolation of LAB. Organic oranges were preferred to increase the possibility of isolating functional LAB, given the absence of synthetic chemicals and pesticides. Initially, distinct colonies were isolated, and microscopic examination was conducted to select only those bacteria exhibiting a rod-shaped morphology. Four different isolates were subsequently identified at the taxonomic level through 16S rRNA gene sequencing. The sequences were compared using the BLAST tool, which revealed a 98% identity with the *Lactiplantibacillus plantarum* species. Furthermore, multiple sequence alignment of the 16S rRNA gene sequences among the isolates indicated a maximum sequence homology of 98%, suggesting that the four isolated strains are distinct strains within the *L. plantarum* species. The nucleotide sequences of the 16S rRNA genes of the isolated strains have been deposited in the GeneBank database under accession numbers PQ358527 for the BEA strain, PQ358528 for the BEA1 strain, PQ358529 for the BIA strain, and PQ358530 for the BIA1 strain, respectively.

### 3.2. Antibacterial Activity of the Isolated Strains and Selection of L. plantarum BIA Strain

The probiotic properties of lactobacilli are known to be strain-dependent, making the selection of strains with strong antibacterial activity crucial. In this study, we evaluated the ability of the isolated *L. plantarum* strains to inhibit the growth of pathogens responsible for infectious diseases in the respiratory tract. Specifically, *Staphylococcus aureus*, which is associated with a broad spectrum of infections affecting the upper respiratory tract [35], as well as *Streptococcus pyogenes,* which colonizes the throat and skin, may cause severe disease and are also the most common bacterial cause of pharyngotonsillitis [36], alongside *Pseudomonas aeruginosa*, which is one of the main pathogens of nosocomial infections [37], and *Escherichia coli* and *Bacillus subtilis*, which are common infectious bacteria.

**Figure 1:**
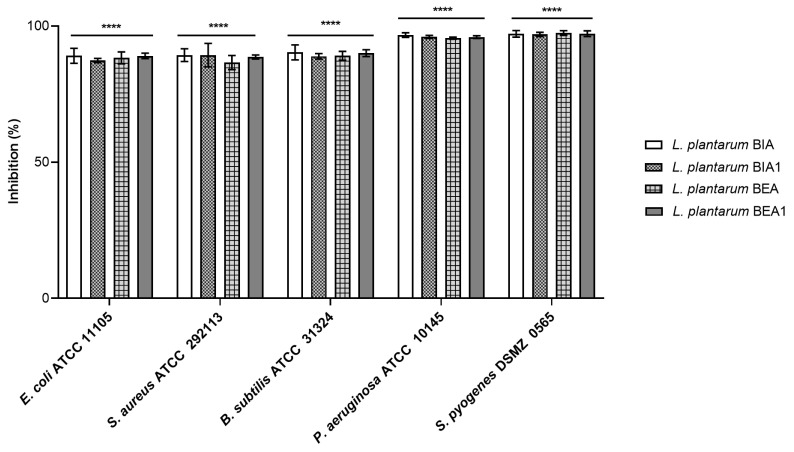
Inhibition of pathogens’ planktonic growth by CFS recovered from *L. plantarum* strains BIA, BIA1, BEA, and BEA1. The pathogens tested were *E. coli* ATCC 11105, *S. aureus* ATCC 292113, *B. subtilis* ATCC 31324, *P. aeruginosa* ATCC 10145, and *S. pyogenes* DSMZ 0565. The % of inhibition was assessed by comparing the turbidity (OD600) of the sample with the untreated control. Data are shown as mean ± SD, n = 3. Significance indicated by **** = *p* < 0.0001 compared to untreated control.

As illustrated in Figure 1, CFS recovered from *L. plantarum* strains exhibited strong inhibitory activity against the pathogens, with slight variations in the percentage of inhibition among the different CFS. CFS from *L. plantarum* BIA strain demonstrated the most effective inhibition of pathogen growth, with inhibition rates of 89.03%, 89.2%, 90.02%, and 96.66% against *E. coli, S. aureus, B. subtilis*, and *P. aeruginosa,* respectively. An exception was noted for *S. pyogenes,* for which the CFS from the *L. plantarum* BEA strain showed a slightly higher inhibition rate (97.4%) compared to that exerted by BIA CFS (97.1%).

The antimicrobial activity of viable *L. plantarum* BIA, BIA1, BEA, and BEA1 cells was also evaluated using an overlay assay. These strains displayed varying degrees of inhibitory effects; for instance, *L. plantarum* BIA was the most active against *P. aeruginosa*, with an inhibition zone measuring between 30 and 32 mm, while BEA showed the strongest activity against *S. pyogenes*, with an inhibition zone ranging from 20 to 24 mm (Table 2). These findings are consistent with previous studies in which various *L. plantarum* strains demonstrated significant antimicrobial activity; for example, nanoparticles containing *L. plantarum* K8 have been reported to suppress *Staphylococcus aureus* respiratory infections and prevent a progression to chronic disease [38], and *L. plantarum* Lp 115 SD-5209 showed the ability to prevent the formation of *Streptococcus pyogenes* biofilms by producing a glycolipid bioactive compound [39].

The inhibitory activity observed for both CFS and viable cells demonstrated that all *L. plantarum* strains were capable of significantly inhibiting pathogen growth. However, combining the results of these two tests, the BIA strain exhibited the strongest overall effect. Consequently, the *L. plantarum* BIA strain was selected to be further characterized as a probiotic candidate for the treatment of respiratory tract infections.

### 3.3. Anti-Inflammatory Activity of L. plantarum BIA

**Figure 2:**
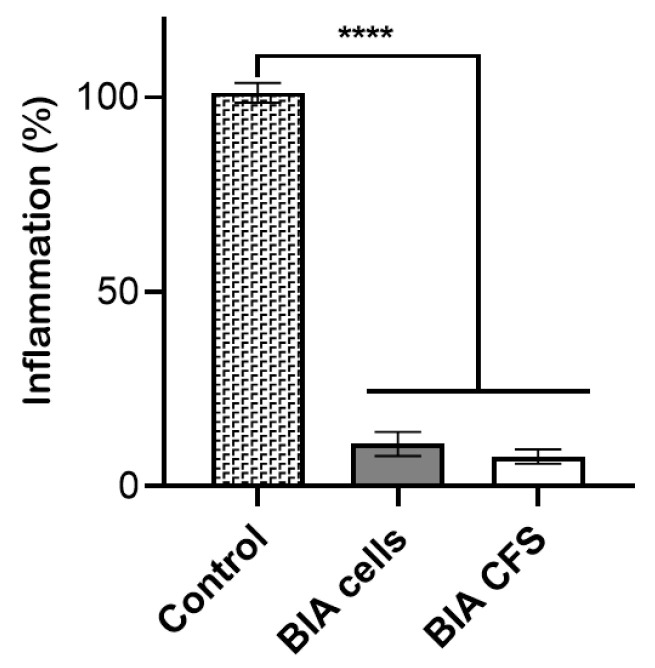
Anti-inflammatory activity of *L. plantarum* BIA cells and CFS. Nitrite production is expressed as % of inflammation. RAW264.7 cells were either stimulated with LPS alone (control, 100%) or stimulated with LPS and treated with *L. plantarum* BIA cells and CFS. Data are shown as mean ± SD, n = 2. Significance indicated by **** = *p* < 0.0001 between samples indicated by the brackets.

Since one of the key contributors to respiratory disease is the heightened inflammatory response caused by damaged or dead microbial pathogens and host cells [40], we sought to investigate the anti-inflammatory potential of the BIA strain. The RAW264.7 cell line, derived from murine macrophages, is widely employed to investigate immune response, particularly in the context of inflammation and infection. Since LPS can activate macrophages and stimulate the secretion of several pro-inflammatory cytokines, including tumor necrosis factor-alpha (TNF–α) and interleukin-1 beta (IL–1β), RAW264.7 macrophages treated with lipopolysaccharide (LPS) are used as a positive control of immune response activation [41]. We performed the Griess reaction assay to estimate nitric oxide (NO) production, a key mediator of inflammation, produced after stimulation of macrophages with LPS. RAW264.7 cells were stimulated with LPS to induce inflammation and then treated with CFS from *L. plantarum* BIA or the corresponding viable cells. As shown in Figure 2, a marked reduction in the percentage of inflammation was observed after both treatments; the residual inflammation was significantly lower compared to the control, corresponding to 10.0% after treatment with the viable cells and 7.0% after treatment with the CFS. These data suggest that CFS and viable cells of *L. plantarum* BIA inhibit the production of NO, thereby reducing inflammation. According to the literature, the potential mechanisms by which *L. plantarum* regulates immunity in the respiratory tract vary among species and strains; some studies have shown that components of *Lactobacillus* can be recognized by pattern recognition receptors in the respiratory epithelium, activating downstream immune pathways [42]. For example, the peptidoglycan from *L. rhamnosus* CRL1505 has been found to enhance the TLR3/RIG-I-triggered antiviral immune response by increasing IFN-γ production and NK cell activity, thereby promoting viral clearance and reducing lung tissue damage [43]. Similarly, protection and prolonged survival against the lethal pneumonia virus infection in MyD88 gene-deleted mice was conferred by administration of *Lactobacillus plantarum* NCIMB 8826 and *Lactobacillus reuteri* F275, suggesting that the protective mechanisms may be TLR-independent [8]. In another study, *Lactobacillus plantarum* DP189 downregulated pro-inflammatory cytokines, such as TNF-α, IL-6, and IL-1β, in models of neurodegenerative diseases, thereby reducing inflammatory responses [44]. Our data, together with findings from the literature, support the hypothesis that the strain *L. plantarum* BIA could be utilized in a nasal spray as a probiotic for the treatment and prevention of infections. Hence, BIA would be advantageous because it inhibits pathogens’ growth and colonization while also modulating inflammation, which is frequently observed in several respiratory diseases. Furthermore, the intranasal administration of *Lactobacillus* has been shown to induce a stronger respiratory immune response compared to oral administration [45,46].

### 3.4. Genome Features of L. plantarum BIA and Safety Profile

The genome sequence of *L. plantarum* BIA was obtained and submitted to NCBI GenBank under accession number CP170649. The strain contains a circular 3,238,827 bp chromosome, encoding approximately 3183 proteins, with a CG content of 44.6%. The annotation of the *L. plantarum* BIA genome revealed the presence of several genes involved in the production of bioactive metabolites including bacteriocins, i.e., antibacterial peptides ribosomally synthesized. In *L. plantarum* species, bacteriocins are known as plantaricins. Analysis of the *L. plantarum* BIA genome identified the genes *plnE, plnF, plnJ,* and *plnK*, which are organized in an operon responsible for the production of two two-peptide bacteriocins, specifically PlnEF and PlnJK; this arrangement is consistent with the findings of Anderssen et al. in *L. plantarum* C11 [47]. The regulation of plantaricin operon is mediated by a signal transduction system, with a histidine protein kinase (HPK) functioning as an environmental sensor. For example, in *L. plantarum* C11, the regulatory operon includes *plnA*, encoding a bacteriocin-like peptide, and *plnB*, encoding the HPK [48]. In the *L. plantarum* BIA genome, *plnA* and *plnB* genes were also found and this suggests that the activation of *plnEF* and *plnJK* is dependent on PlnA, which interacts with HPK PlnB to regulate bacteriocin production. The presence of plantaricin operons in the BIA strain genome supports the strong antimicrobial activity described in Section 3.2. The genome annotation also revealed the presence of the *dltB* gene, which encodes for teichoic acid D-alanyltransferase. Teichoic acid is a fundamental component of the cell wall in Gram-positive bacteria and plays a key role in interacting with TLR2; this gene was identified as responsible for immunomodulatory effects, as reported in the study from O’Shea (2013) [49]. The *L. plantarum* BIA genome was also searched for the presence of genes related to virulence and antibiotic resistance by using different tools. AMRFinderPlus detected the c*lpL* gene, which encodes for caseinolytic protease L (ClpL). ClpL is a chaperonine, acting by refolding denatured proteins into their correct conformation and facilitating the proteolysis of damaged proteins, thereby reducing denatured proteins in an ATP-dependent manner [50]. In some pathogen strains, ClpL has been associated with virulence and stress resistance; however, ClpC, a protein belonging to the same class as ClpL, was identified in the genome of safe strains and described as a probiotic marker. As an example, ClpC and ClpL were found in various *L. plantarum* strains, including PA21, and associated with physiological stress tolerance, crucial for bile and acid tolerance [51,52]. Additionally, RGI-CARD detected the presence of the *qacJ* gene, which encodes for an efflux pump that confers resistance to quaternary ammonium compounds, employed as disinfectants and antiseptics agents. This gene is classified within the small multidrug resistance (SMR) family; it is commonly found in other *L. plantarum* strains, such as *L. plantarum* 18 and *L. plantarum* BRD3A [53,54]. The analysis conducted with ResFinder tool did not reveal any genes associated with antibiotic resistance and virulence.

The sequencing of *L. plantarum* BIA DNA also revealed the presence of four natural plasmid-related sequences. As reported by numerous studies, plasmids are quite common in *L. plantarum* species, and generally bear genes involved in bacteriocins and exopolysaccharides synthesis [55,56]. The annotation and the AMRFinder analysis exclude the presence of virulence and antibiotic resistance genes; at the same time, no genes directly involved in bacteriocins production were identified. We can thus conclude that *L. plantarum* BIA can be considered safe from the genetic point of view.

### 3.5. Selection of the Lyophilization Media

Converting probiotics into a dried form through vacuum freeze-drying is a suitable strategy to maintain their viability and properties over storage and transport [57]. However, this technological process subjects cells to various stresses, including cold stress, crystallization, osmotic pressure, and desiccation, which can strongly affect cell survival by negatively impacting membrane integrity, cell metabolism, and enzymatic activity [57,58]. As the composition of the medium is closely linked to the bacteria survival following freeze-drying, the issue can be tackled by providing the microorganisms with those substrates required for their survival. Usually, lyophilization media (LM) contain some or all these components: carbon source, nitrogen source, and cryoprotectants [59]. Carbohydrates, like disaccharides (sucrose, trehalose, lactose), polyols (mannitol, sorbitol), polysaccharides (maltodextrin, dextran, inulin), and oligosaccharides, are frequently exploited for their double function: on the one hand, they are a source of nutrients, and on the other hand, they increase the glass transition temperature (T_g_) and maintain the natural structure of membrane proteins [60]. Even tryptone, casein peptone, or yeast extract can be employed as substrates to sustain cell survival, as they provide amino acids and vitamins. Moreover, thanks to its ability to increase the T_g_ and, at the same time, layer the cell membrane with a protective coating that limits osmotic pressure differences, skim milk (SK) has been widely exploited as a cryoprotectant [59].

**Figure 3:**
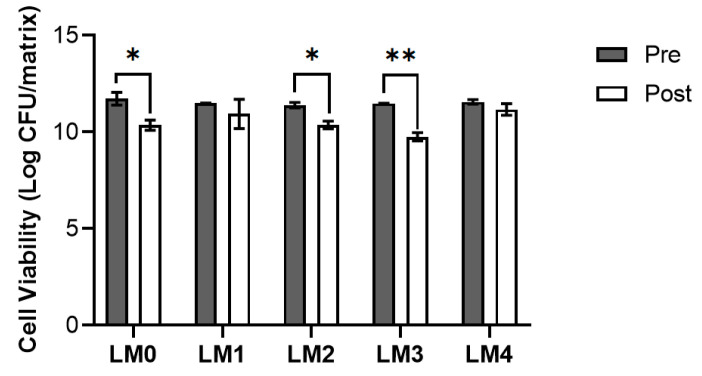
Influence of different lyophilization media (LM) on *L. plantarum* BIA cell viability before (Pre) and after (Post) freeze-drying. Media composition: 10% SK, 0.1% AA, 0.5% LCT, 0.5% YE for LM0; 10% FOS, 0.1% AA, 0.5% LCT, 0.5% YE for LM1; 10% IN, 0.1% AA, 0.5% LCT, 0.5% YE for LM2; 10% FOS, 0.1% AA, 0.5% LCT, 0.5% IN for LM3; 10% FOS, 0.1% AA, 0.5% LCT for LM4. Data are reported as mean ± SD, n = 2. Significance indicated by * = *p* < 0.05; ** = *p* < 0.01 between samples indicated by the brackets.

To identify the LM capable of protecting probiotic cells during freeze-drying and preserving their viability over storage, a well-established medium previously used in our laboratory as a generic medium for lactobacilli lyophilization, here named LM0, was used as the reference. The freeze-drying of LM0 resulted in a compact matrix and, as depicted in Figure 3, its employment did not prevent a one-fold decrease in viability; moreover, when hydrated with the lead polymeric solution (see Section 3.6 and Section 3.7), the reconstitution required more than 20 min and heavy stirring, which would be inconvenient for the patient. Therefore, LM0 was modified by replacing SK with fructooligosaccharides (FOS) or inulin (IN), obtaining LM1 and LM2, respectively, and the combination of the two was also tested, resulting in LM3. Despite the change in composition, the lyophilization of LM1–LM3 produced matrices, but only LM1 could maintain cell viability almost unchanged after freeze-drying, and upon reconstitution with the lead polymeric solution, its dissolution took place in a few seconds: as the freeze-dried matrix encountered the polymer, it immediately dissolved. Even though LM1 outperformed the other tested LM, considering the nasal application of the final product, it was further optimized to address the issue of the unpleasant smell due to the presence of yeast extract (YE). Interestingly, LM4 resembled LM1 in terms of both appearance and performance, meaning that YE removal from the medium did not significantly (*p* > 0.05) affect cell viability. Therefore, LM1 and its optimized composition, LM4, were selected as the candidate media.

### 3.6. Characterization of Polymer Solutions

A considerable aspect of nasal products is a pH as close as possible to the physiological one. In fact, medications with pH values between 5 and 6.5 are generally believed to be unable to induce inflammation or irritation of the nasal mucosa [61]. In addition, a key challenge associated with nasal delivery is the rapid removal of formulations from the nasal mucosa due to mucociliary clearance. The latter represents a defense mechanism against pollutants, allergens, and xenobiotics. Specifically, it results from the combination of the barrier effect of the mucous layer and its movement from the anterior to the posterior area of the nasal cavity, enabled by the cilia beating of ciliated epithelial cells [62]. Consequently, formulating strategies must be adopted to prolong the residence time of the nasal product, supporting the colonization of the nasal cavity by the administered probiotic. Two main approaches can be exploited: the use of mucoadhesive polymers or the employment of viscosity enhancers [62]. In the present study, the nasal spray included polymers bearing both abilities: cellulose derivatives, like hydroxypropylmethylcellulose of different viscosities, and hyaluronic acid. The selected polymers were thus tested at various concentrations and characterized in terms of pH, viscosity, and mucoadhesive potential in parallel. The first screening was performed only on polymeric solutions, from here on named “plain” polymeric solutions, to evaluate whether it was possible to identify a lead molecule to be used at a preferred concentration.

The nasal spray object of this research will contain viable probiotic cells, and since *L. plantarum* BIA is a lactic acid bacterium, it is reasonable to think that it would cause a slight acidification of the resuspending medium. Therefore, polymers were dissolved in PBS at pH 6.8 rather than in saline solution or water to prevent a possible drop in the pH value of the final product obtained upon reconstitution of the freeze-dried BIA cells. As expected, all tested plain solutions exhibited a pH of 6.50–7.00.

**Figure 4:**
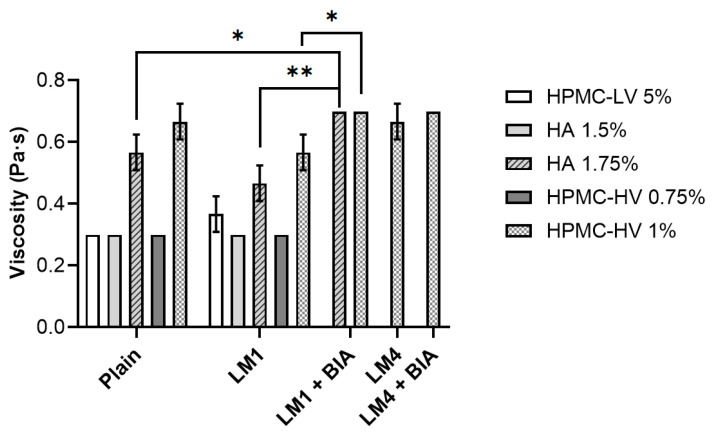
Viscosity of the different polymer solutions. Reported values refer to the sole polymer solution (plain) or the polymer solution once used to resuspend the freeze-dried matrices made of LM1 (10% FOS, 0.1% AA, 0.5% LCT, 0.5% YE) or LM4 (10% FOS, 0.1% AA, 0.5% LCT) components, in the absence or presence (LM1/LM4+BIA) of *L. plantarum* BIA cells. Data are reported as mean ± SD, n = 3. Significance indicated by * = *p* < 0.05; by ** = *p* < 0.01 between samples indicated by the brackets.

Figure 4 shows that focusing on the lowest concentration tested for HPMC-LV, HA, and HPMC-HV, all plain solutions exhibited the same viscosity, equal to 0.30 ± 0.00 Pa·s, which was already higher than that of nasal drops or nasal solutions usually employed for nasal irrigation or decongestion. Nevertheless, Patterlini and co-workers performed some characterization studies, including viscosity measurements, on commercially available nasal sprays (Ryaltris, Dymista, Nasonex, Avamys), demonstrating that the tested products featured viscosities between 25 and 130 Pa·s [63]. Therefore, the data suggest that a higher concentration of polymers can be used. Alongside this, the study aimed to develop a freeze-dried probiotic to be delivered as a nasal spray upon reconstitution, and thus the administered product represents a suspension. It is reported that an increased viscosity is beneficial for pharmaceutical suspensions as the viscosity of the suspending medium will reduce the phenomenon of particulate sedimentation, allowing accuracy of dosing [64]. Because a viscosity of 0.3 Pa·s was reached by HPMC-LV only when using it at 5%, this meant that the polymer should have been used at significantly higher concentrations compared to HA and HPMC-HV to observe a noticeable increase in the sample viscosity. Consequently, viscosity rise upon polymers’ concentration increase was determined only for HA and HPMC-HV. HA 1.75% and HPMC-HV 1% reached values of 0.57 ± 0.06 Pa·s and 0.67 ± 0.06 Pa·s, respectively, without being statistically different (*p* > 0.05).

**Figure 5:**
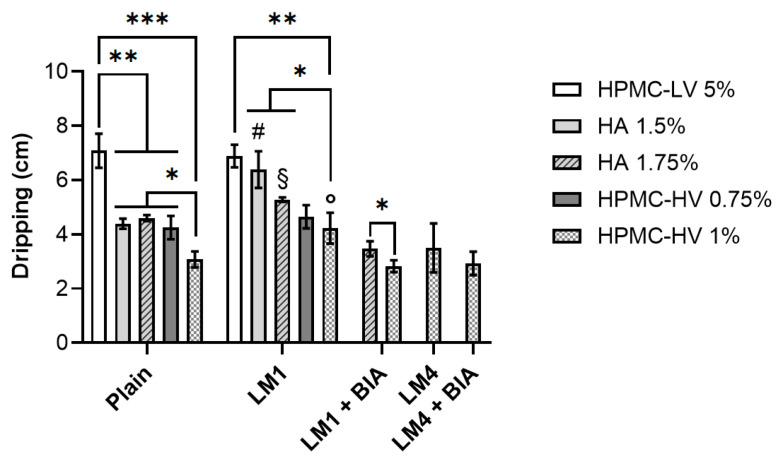
Dripping ability of the different polymer solutions. Reported values refer to the sole polymer solution (plain) or the polymer solution once used to resuspend the freeze-dried matrices made of LM1 (10% FOS, 0.1% AA, 0.5% LCT, 0.5% YE) or LM4 (10% FOS, 0.1% AA, 0.5% LCT) components, in the absence or presence (LM1/LM4+BIA) of *L. plantarum* BIA cells. Data are reported as mean ± SD, n = 3. Significance indicated by * = *p* < 0.05; by ** = *p* < 0.01; by *** = *p* < 0.001 between samples indicated by the brackets. Significance indicated by # = *p* < 0.05 compared to HA 1.5% plain solution; by § = *p* < 0.05 compared to HA 1.75% plain solution; by ° = *p* < 0.05 compared to HPMC-HV 1% plain solution.

The lack of significant differences in terms of viscosity values between plain solutions when polymers were used at lower or higher concentrations enabled us to point out the diverse mucoadhesive potential exclusively based on the polymers’ chemical properties. First, the dripping test was performed, and it was exploited because it offers the chance to investigate both excipients’ adhesive ability and the tendency of the nasal product to drip off after administration [63]. Considering plain solutions, Figure 5 shows that, once applied on the functionalized paper-based filter, HPMC-LV 5% dripped for 7.08 ± 0.63 cm, thus being displaced over a remarkably higher distance (*p* < 0.05) compared to all the other tested samples. The inferior mucoadhesive ability of HPMC-LV compared to HA and HPMC-HV plain solutions, despite the higher polymer content of its sample, supported our previous choice of not investigating the use of HPMC-LV at increased concentrations. A different behavior was observed for HA and HPMC-HV depending on the polymers’ concentration. The use of HA 1.75% did not result in a significant decrease (*p* > 0.05) in the dripping of the plain solution compared to HA 1.5% (4.39 ± 0.18 cm for HA 1.5% and 4.60 ± 0.11 cm for HA 1.75%). Conversely, HPMC-HV 1% determined a relevant reduction (*p* < 0.05) in the displacement distance compared to HPMC-HV 0.75% (4.25 ± 0.43 cm for HPMC-HV 0.75% and 3.08 ± 0.31 cm for HPMC-HV 1%). Furthermore, even though no significant differences (*p* > 0.05) could be seen between HA 1.5% and HPMC-HV 0.75%, the use of HPMC-HV 1% resulted in the shortest dripping distance compared to all other tested samples (*p* < 0.05). While performing the dripping analysis, it was noted that further increases in HA and HPMC-HV concentrations would have clogged the spray nozzle, preventing proper and easy administration of the product.

**Figure 6:**
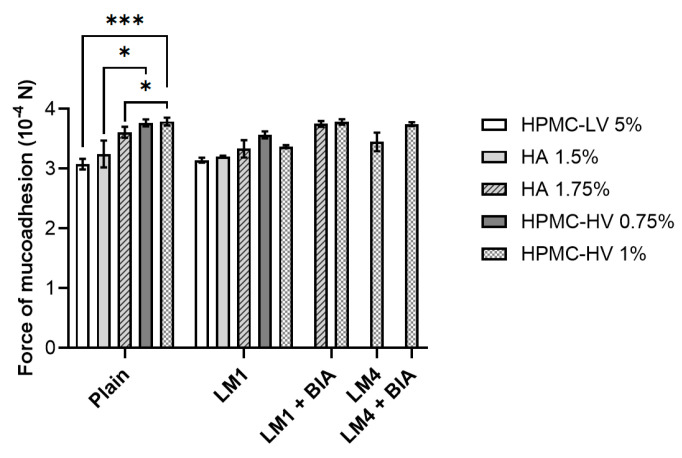
Force of mucoadhesion measured for the different polymer solutions. Reported values refer to 3 mL of the sole polymer solution (plain) or the polymer solution once used to resuspend the freeze-dried matrices made of LM1 (10% FOS, 0.1% AA, 0.5% LCT, 0.5% YE) or LM4 (10% FOS, 0.1% AA, 0.5% LCT) components, in the absence or presence (LM1/LM4+BIA) of *L. plantarum* BIA cells. Data are reported as mean ± SD, n = 3. Significance indicated by * = *p* < 0.05; by *** = *p* < 0.001 between samples indicated by the brackets.

The adhesive properties of the resuspending media were further investigated by determining the force of mucoadhesion. Overall, data reported for plain solutions in Figure 6 confirmed what had been observed during the dripping test. Hence, the weakest force was measured while detaching the nasal mucosa from HPMC-LV 5% (3.08 ± 0.09 10^−4^ N), whereas the highest force of mucoadhesion was determined for HPMC-HV 1% (3.79 ± 0.07 10^−4^ N). Contrary to what was observed for the dripping test, moving from 0.75% (*w*/*v*) of HPMC-HV to 1% (*w*/*v*) did not allow for a mucoadhesiveness improvement (*p* > 0.05). However, regardless of the concentration, HPMC-HV was more mucoadhesive (*p* < 0.05) than HA-containing plain solutions.

This first screening procedure on plain polymer solutions revealed that HPMC-HV 1% was the best-performing resuspending medium as it was more viscous than a conventional liquid formulation for nasal delivery and showed a reduced tendency to drip off after administration because of a superior ability to interact with the mucus layer, as confirmed by the force of mucoadhesion test.

### 3.7. Characterization of Polymer Solutions upon Reconstitution of the Freeze-Dried Matrix

Since the plain polymer solution will be used to reconstitute the lyophilized probiotic, it was necessary to investigate whether and to what extent the components of the lyophilization media (LM) and the bacterial cells could alter pH, viscosity, and mucoadhesion.

Because LM1 was the first lyophilization media to be identified and it was more complex than the optimized LM4, eventual changes in polymer solutions’ features were initially investigated in the presence of the freeze-dried LM1 without cells (LM1 or LM4 in Figure 4, Figure 5 and Figure 6). The use of plain solutions to resuspend LM1 resulted in products that all featured a pH of 6. Concerning viscosity (Figure 4), the trend of the data closely resembled that obtained with plain solutions. However, non-statistically significant differences (*p* > 0.05) could be observed for HPMC-LV as well as for HA and HPMC-HV at the highest concentration tested, compared to the respective plain solutions. When looking at Figure 5, and specifically at LM1 data, it can be noted that the presence of LM1 caused a general increase in the dripping compared to plain solutions, which was significantly different for HPMC-HV 1% and HA at both concentrations tested. Nevertheless, the overall outcome of the dripping analysis remained unchanged even in the presence of LM1: the longest dripping distance was achieved by HPMC-LV 5% (6.89 ± 0.42 cm) which, unlike HPMC-HV 1%, reduced the dripping to 4.23 ± 0.57 cm, covering a distance that was remarkably shorter than those of HPMC-LV and HA. Similarly, the force of mucoadhesion of LM1 samples also followed the same pattern seen with plain solutions (Figure 6). Nonetheless, it was unexpected to find that in the presence of LM1 components, no significant differences (*p* > 0.05) could be observed between HA and HPMC-HV. Altogether, these results suggested that the viscosity reduction, which was probably due to a different organization of the polymers’ network because of the presence of the LM’s excipients, caused a decrease in the polymer solutions’ mucoadhesive ability. Hence, when used to reconstitute the freeze-dried LM1, the resulting formulation tended to drip more easily along the functionalized paper filter, and less force was necessary to separate the nasal mucosa from the formulations.

The second screening procedure demonstrated that when the lead plain solution HPMC-HV 1% was used to resuspend the freeze-dried LM1, its viscosity-enhancing effect was slightly reduced, and it performed simultaneously better than HPMC-LV and HA (at both concentrations) only in terms of dripping behavior. Consequently, the influence of LM1-containing BIA cells (LM1+BIA) on pH, viscosity, and mucoadhesion was evaluated only on HA 1.75% and HPMC-HV 1%. As predicted, the presence of BIA cells caused a reduction in the pH of the final products, which were characterized by pH values of 5.50–5.00. In the case of HA 1.75%, the formation of the cell suspension resulted in a significant (*p* < 0.05) increase in viscosity (0.70 ± 0.00 Pa·s) compared to both its respective plain solution and LM1 (0.47 ± 0.06 Pa·s) samples (Figure 4). A slight increase was also seen for HPMC-HV 1%, whose viscosity reached 0.7 ± 0.00 Pa·s, thus being close to the viscosity of the plain solution and the same as HA 1.75%+LM1+BIA (Figure 4). Despite being characterized by the same viscosity, the dripping test performed on the selected polymer solutions in the presence of the freeze-dried BIA in LM1 (LM1+BIA) displayed the superior tendency of HA 1.75% (3.47 ± 0.28 cm) compared to HPMC-HV 1% (2.83 ± 0.22 cm) to drip off the mucin-coated paper filter (Figure 5). Moreover, Figure 5 shows that the interaction between HPMC-HV 1% and the mucus layer was completely unaffected by the final composition (LM excipients and BIA cells) of the freeze-dried matrix. Nevertheless, in LM1+BIA samples, the force of mucoadhesion was not significantly different (*p* > 0.05) between HA 1.75% and HPMC-HV 1% (Figure 6).

Based on the results from the third screening, the dripping ability was the only distinguishing feature that could guide the selection of the polymer and its preferred concentration. Therefore, HPMC-HV 1% was identified as the lead resuspending solution and employed to reconstitute the optimized lyophilization media, LM4, in the absence and presence of BIA (LM4 and LM4+BIA samples, respectively). The samples’ patterns of pH, viscosity, and mucoadhesion overlapped with those of HPMC-HV 1% in the case of LM1 and LM1+BIA. Compared to the HPMC-HV 1% plain solution, viscosity values were never significantly different (*p* > 0.05) (Figure 4), the displacement distance during the dripping test was slightly increased in the LM4 sample (3.50 ± 0.90 cm) but was perfectly restored when the LM4 contained the probiotic (2.93 ± 0.43 cm) (Figure 5), and the same was true for the force of mucoadhesion (3.45 ± 0.16 10^−4^ N for LM4; 3.75 ± 0.03 10^−4^ N for LM4+BIA) (Figure 6).

### 3.8. Nasal Spray Shelf Life and Probiotic’s Biological Activities upon Formulation

**Figure 7:**
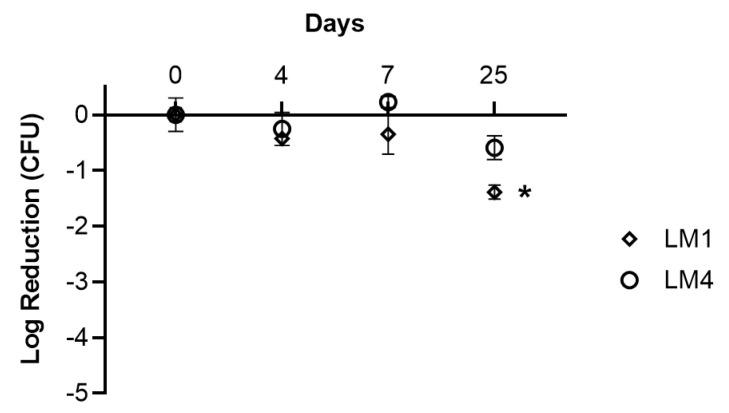
Stability of *L. plantarum* BIA formulated as a nasal spray: freeze-dried in LM1(10% FOS, 0.1% AA, 0.5% LCT, 0.5% YE) or LM4 (10% FOS, 0.1% AA, 0.5% LCT) and resuspended in HPMC-HV 1%. Cell viability was tested at the time of reconstitution (day 0) and after 4, 7, and 25 days. Data are reported as mean ± SD, n = 2. Significance indicated by * = *p* < 0.05 compared to LM1 on day 0.

The selected polymeric solution, HPMC-HV 1%, was used to reconstitute the lead freeze-dried matrices LM1 and LM4 containing *L. plantarum* BIA cells, aiming to determine the maximum shelf life of the reconstituted product when stored at +4–8 °C. Since the functional properties of the nasal spray are linked to BIA cell viability, probiotic survival was the first evaluated feature. The cell viability profiles in the presence of LM1 or LM4, shown in Figure 7, remained constant and overlapped until day 7, then differed on day 25 when both samples featured a decrease compared to the respective zero-time point. However, such a decrease was statistically significant just for the LM1 sample, revealing that the absence of YE in LM4 did not negatively affect BIA survival. The obtained stability profiles are in line with the shelf life of the commercially available nasal spray Rinogermina^®^ and with the data reported in other research papers. Specifically, Jokicevic and co-workers (2021) developed a spray-dried powder containing *L. casei* AMBR2 cells to be reconstituted as a nasal spray. These authors studied the effect of different disaccharide or sugar alcohol combinations on the stability of the product and observed that the decrease in cell viability began after 48h, losing half-Log or a Log after 7 days from the reconstitution [3]. Moreover, focusing on cell viability data on day 7 and considering that the average puff of the nasal spray is 0.2 mL, the formulation containing LM1 can deliver more than 5 × 10^8^ CFU/puff, and that with LM4 more than 1 × 10^9^ CFU/puff. Both values are in agreement with previously published data on nasally administered probiotics [65,66]. Alongside, more batches were prepared to verify the reconstituted products’ stability in terms of pH and viscosity within 7 days. After one week of storage, for both LM1 and LM4, viscosity values remained equal to 0.70 ± 0.00 Pa s, while the pH decreased to 4.50–5.00. pH reduction upon storage of the reconstituted product was also reported by Jokicevic et al. (2021) and is likely due to the metabolic activity of the probiotic: BIA cells did not increase in number but continued to release metabolites, such as lactic acid, that contributed to the acidification of the media. Nevertheless, the nasal spray can be safely administered into the nasal cavity, as it has been reported that the mucosal tissue remains undamaged as long as the pH stays between 4 and neutral values [61].

**Figure 8:**
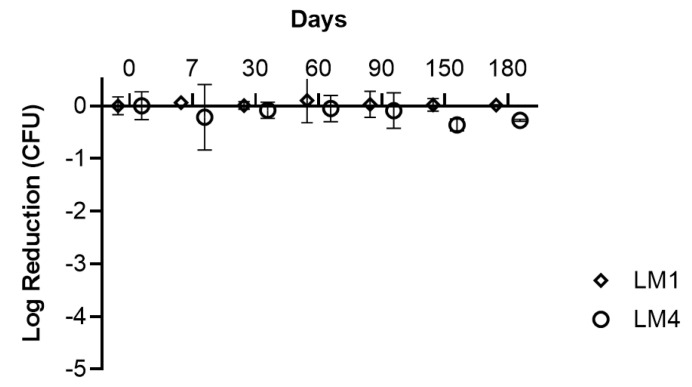
Stability of *L. plantarum* BIA after freeze-drying. Viability of BIA cells freeze-dried in LM1(10% FOS, 0.1% AA, 0.5% LCT, 0.5% YE) or LM4 (10% FOS, 0.1% AA, 0.5% LCT) was tested immediately after lyophilization (day 0) and after 7, 30, 60, 90, 150, and 180 days.

When it comes to reconstituted dosage forms, a complete overview of the stability should include the effect of storage conditions on its single components. In fact, the product must stay in an inactive state for an extended period and reactivate when rehydrated [67]. In this study, the survival rate of BIA cells was evaluated in LM1 and LM4 matrices after 6 months of storage at +4–8 °C following the lyophilization process. As shown in Figure 8, cell viability was assessed immediately after freeze-drying and at 7, 30, 90, 150, and 180 days post-lyophilization. The results indicate that after 6 months, the survival rate of BIA cells remained stable in both matrices, suggesting that the presence of YE in LM1 is not strictly required to sustain cell viability. Furthermore, the product maintained a high survival rate, assuring that the reconstituted product preserves its properties even after six months. The same was verified for the polymeric solution, which featured a viscosity of 0.70 ± 0.00 Pa s and a pH in the range of 6.50–7.00 during the same span.

Considering that the formulation process subjects the probiotic to relevant stresses, namely freezing and dehydration, and that the reconstituted product includes a variety of excipients, it was necessary to evaluate whether these factors could influence not only the survival rate but also the probiotic’s biological activity. Therefore, the effect of lyophilization and reconstitution on antimicrobial activity was investigated. The antagonistic activity of *L. plantarum* BIA cells freeze-dried in LM1 or LM4 matrices was evaluated when reconstituted after 0 days and 90 days from the lyophilization process, and results are shown in Table 3. Overall, regardless of the composition of the lyophilization media, *L. plantarum* BIA demonstrated noteworthy antimicrobial properties towards all the tested pathogens, and only slight differences were observed in comparison to non-formulated BIA cells (see Section 3.2). Since the genome analysis pointed out that *L. plantarum* BIA can produce plantaricins, it is reasonable to suppose that the dried preparation maintained plantaricin production ability, as hypothesized by Kamil et al. (2020) and Silva et al. (2002) [68,69].

**Figure 9:**
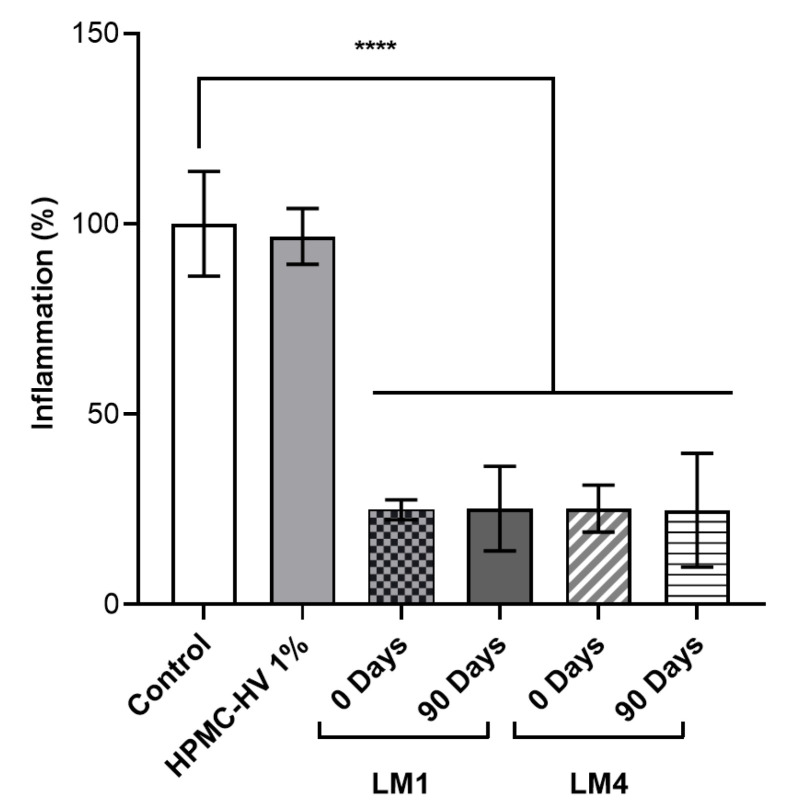
Anti-inflammatory activity of *L. plantarum* BIA upon formulation. Nitrite production is expressed as % of inflammation. RAW264.7 cells were either stimulated with LPS alone (control, 100%) or stimulated with LPS and treated with *L. plantarum* BIA cells, freeze-dried in LM1 (10% FOS, 0.1% AA, 0.5% LCT, 0.5% YE) or LM4 (10% FOS, 0.1% AA, 0.5% LCT) and reconstituted in HPMC-HV 1%, at 0 and 90 days after the lyophilization process. Data are shown as mean ± SD, n = 2. Significance indicated by **** = *p* < 0.0001 between samples indicated by the brackets.

Once it was confirmed that the stability of cell viability goes along with the antimicrobial activity, the maintenance of the anti-inflammatory activity was also evaluated for freeze-dried *L. plantarum* BIA reconstituted with HPMC-HV 1% at 0 and 90 days after the freeze-drying process. As depicted in Figure 9, when the strain was freeze-dried and immediately reconstituted (0 days), *L. plantarum* BIA formulated in LM1 and LM4 reduced the inflammation to 24.8% and 25.2%, respectively. Similarly, when *L. plantarum* BIA was reconstituted 90 days post-lyophilization, the freeze-dried strain formulated in LM1 and LM4 reduced the inflammation to 24.7% and 25.2%, respectively. These findings suggest that the presence of YE in the lyophilization medium does not lead to significant differences in the anti-inflammatory activity. Additionally, it was demonstrated that the probiotic function remained unchanged during 3 months of storage because the reduction in inflammation seen at 0 days post-lyophilization was not significantly different from the reduction seen at 90 days. However, following freeze-drying and reconstitution, anti-inflammatory activity decreased compared to non-formulated *L. plantarum* BIA cells. Given that the anti-inflammatory activity is attributed to the presence of surface molecules likely involved in inflammatory processes [8], we hypothesized that the components of the lyophilization media may mask these effectors, leading to a reduction in anti-inflammatory activity. However, despite this reduction, *L. plantarum* BIA remains able to reduce NO release upon LPS stimulation. These results are in line with the study from Layus, B.I (2022), in which *L. plantarum* CRL 759 maintained its anti-inflammatory activity up to 30 days of storage [70].

## 4. Conclusions

The nasal delivery of probiotics with antimicrobial and anti-inflammatory properties offers a promising alternative to traditional treatments for respiratory infections, namely otitis media, chronic rhinosinusitis, and acute sinusitis, especially in the context of rising antibiotic resistance. Through careful formulation and development, nasal sprays can effectively deliver viable probiotic cells to the nasal cavity, providing a preventive and therapeutic approach to maintaining respiratory health. The developed formulation consists of the probiotic-containing freeze-dried matrix, which is made of 10% fructooligosaccharides, 0.1% ascorbic acid, and 0.5% lactose, and the resuspending medium, i.e., the HPMC-HV 1% polymer solution. The reconstituted product features mucoadhesive properties, as demonstrated by the short dripping distance (2.93 cm) and the force of mucoadhesion (equal to 3.75 10^−4^ N), a suitable shelf-life of a week, and noteworthy anti-inflammatory (inflammatory reduction to 25.2%) and antimicrobial activity against relevant pathogens of the upper respiratory tract. Based on these findings, the new strain *Lactiplantibacillus plantarum* BIA administered as a reconstituted mucoadhesive spray could represent a significant advancement in respiratory health management, highlighting the potential of nasal administration of probiotics. Notably, a point of strength of our research is the development of a novel nasal spray formulation containing viable lactobacilli cells from orange peel able to exert both antibacterial and anti-inflammatory functions. To the best of our knowledge, this is the first prototype with these features described in the literature. Future investigations might be focused on the selection of the most suitable device to administer the product to the nasal cavity but also on the deposition profile of the nasal spray using silicon nasal cavity models. Additional studies could also investigate the ability of the nasal formulation to promote probiotic adhesion to relevant nasal epithelial cell lines.

## Figures and Tables

**Table 1:** Composition of the different tested lyophilization media (LM).

Lyophilization Media	Excipients (% *w*/*v*)					
	SK	FOS	AA	LCT	YE	IN
LM0	10		0.1	0.5	0.5	
LM1		10	0.1	0.5	0.5	
LM2			0.1	0.5	0.5	10
LM3		10	0.1	0.5		0.5
LM4		10	0.1	0.5		

SK = skim milk; FOS = fructooligosaccharides; AA = ascorbic acid; LCT = lactose; YE = yeast extract; IN = inulin.

**Table 2:** Antibacterial activity of *L. plantarum* BIA, BIA1, BEA, and BEA1 viable cells against pathogens (overlay assay). The antagonistic activity was expressed as follows: +, radius between 6–14; ++ radius between 14–22 mm; +++, radius > 22 mm.

	*E. coli* ATCC 11105	*S. aureus* ATCC 292113	*B. subtilis* ATCC 31324	*P. aeruginosa* ATCC 10145	*S. pyogenes* DSMZ 0565
*L. plantarum* BIA	++	++	++	+++	++
*L. plantarum* BIA1	++	+	++	+++	++
*L. plantarum* BEA	++	+	++	+++	++
*L. plantarum* BEA1	++	++	++	+++	++

**Table 3:** Antibacterial activity of *L. plantarum* BIA freeze-dried in LM1 (10% FOS, 0.1% AA, 0.5% LCT, 0.5% YE) and LM4 (10% FOS, 0.1% AA, 0.5% LCT) and reconstituted in HPMC-HV 1%, at 0 and 90 days after the lyophilization process. The antagonistic activity showed by *L. plantarum* BIA upon formulation was expressed as follows: -, radius 0–5mm; ++ radius between 14–22 mm; +++, radius > 22 mm.

	Days	*E. coli* ATCC 11105	*S. aureus* ATCC 292113	*B. subtilis* ATCC 31324	*P. aeruginosa* ATCC 10145	*S. pyogenes* DSMZ 0565	HPMC-HV 1%
LM1	0	++	++	++	++	++	-
	90	++	++	++	+++	++	-
LM4	0	++	++	++	+++	+++	-
	90	+++	++	++	+++	++	-

## Data Availability

Data of antimicrobic assays (in vitro antibacterial activity of cell-free supernatants and viable cells) and anti-inflammatory assays (anti-inflammatory activity) of the new strain *Lactiplantibacillus plantarum* BIA as well as the technological characterization and stability of the nasal spray without and with viable BIA (viscosity, mucoadhesion force, dripping test, cell viability) are published in the AMS acta repository (DOI: doi.org/10.6092/unibo/amsacta/7864). The nucleotide sequences of the 16S rRNA genes of the isolated *L. plantarum* strains were submitted to the GeneBank database under accession numbers PQ358527 for BEA strain, PQ358528 for BEA1 strain, PQ358529 for BIA, PQ358530 for BIA1 strain; *L. plantarum* BIA genome was submitted under accession number CP170649.
